# Photonic waveguide to free-space Gaussian beam extreme mode converter

**DOI:** 10.1038/s41377-018-0073-2

**Published:** 2018-10-10

**Authors:** Sangsik Kim, Daron A. Westly, Brian J. Roxworthy, Qing Li, Alexander Yulaev, Kartik Srinivasan, Vladimir A. Aksyuk

**Affiliations:** 1 Center for Nanoscale Science and Technology, National Institute of Standards and Technology, Gaithersburg, MD 20899 USA; 2 Maryland Nanocenter, University of Maryland, College Park, MD 20742 USA; 3 Department of Electrical and Computer Engineering, Texas Tech University, Lubbock, TX 79409 USA

## Abstract

Integration of photonic chips with millimeter-scale atomic, micromechanical, chemical, and biological systems can advance science and enable new miniaturized hybrid devices and technology. Optical interaction via small evanescent volumes restricts performance in applications such as gas spectroscopy, and a general ability to photonically access optical fields in large free-space volumes is desired. However, conventional inverse tapers and grating couplers do not directly scale to create wide, high-quality collimated beams for low-loss diffraction-free propagation over many millimeters in free space, necessitating additional bulky collimating optics and expensive alignment. Here, we develop an extreme mode converter, which is a compact planar photonic structure that efficiently couples a 300 nm × 250 nm silicon nitride high-index single-mode waveguide to a well-collimated near surface-normal Gaussian beam with an ≈160 µm waist, which corresponds to an increase in the modal area by a factor of >10^5^. The beam quality is thoroughly characterized, and propagation over 4 mm in free space and coupling back into a single-mode photonic waveguide with low loss via a separate identical mode converter is demonstrated. To achieve low phase error over a beam area that is >100× larger than that of a typical grating coupler, our approach separates the two-dimensional mode expansion into two sequential separately optimized stages, which create a fully expanded and well-collimated Gaussian slab mode before out-coupling it into free space. Developed at 780 nm for integration with chip-scale atomic vapor cell cavities, our design can be adapted for visible, telecommunication, or other wavelengths. The technique can be expanded to more arbitrary phase and intensity control of both large-diameter, free-space optical beams and wide photonic slab modes.

## Photonic chips: Converting the light

A procedure for converting light beams between widely differing modes will help fulfill the potential of photonic chips - devices that use light for data processing rather than the electrical circuits of conventional microchips. Developing photonic chip technology requires reliable interconversion between the nanometer-scale light beams traveling within the chips and wider beams that can serve as useful output from or input to the devices. Vladimir Aksyuk and colleagues at the National Institute of Standards and Technology, in Gaithersburg, Maryland, USA, have developed a ‘mode converter’ that interconverts the light beams in a photonic chip and external beams that are more than 100,000 times as wide. This reliable and precisely controlled interconversion should assist the development of wide range of applications for photonic chips, including their use in computation, optical analysis and military technologies.

## Introduction

Chip-scale photonic devices have advanced fundamental research in atomic physics^1–4^, time/frequency metrology^5–8^, and biology^9–13^, as well as in industrial applications such as telecommunications^14–16^ and light detection and ranging (LIDAR)^17–19^. In many such applications, efficient coupling of nanophotonic circuits with engineered, application-specific free-space optical fields in millimeter-scale volumes has opened up a broad range of possibilities for chip-scale, highly integrated sensors and systems. For example, the National Institute of Standards and Technology (NIST) is currently implementing chip-scale photonic systems with integrated atomic vapor cavities^1,2^. Realizing the full potential of such systems requires advances in the development of compact, accurate, and efficient optical coupling between sub-micrometer-wide photonic waveguide modes and at least 100 µm wide free-space modes, such as multiple overlapping plane waves and Gaussian and Bessel beams. The main challenge is to decrease the circuit footprint and increase the accuracy of the intensity, phase, and polarization control that are achieved in this extreme mode conversion, which spans multiple orders of magnitude in mode size.

Grating couplers are the most widely known approach to interfacing a photonic mode and a radiation mode;^20–39^ the spatial phase modulation of periodic gratings compensates for the momentum mismatch between the photonic and radiation modes. Since the early 1970s^22–25^, several types of grating structures have been investigated, mainly to couple light from a photonic chip to an optical fiber. In such cases, a compact and highly efficient coupler is desirable and various grating designs have been proposed and demonstrated for various polarizations (TE and/or TM) and spectral bands (C- and O-bands). These couplers do not consider the phase and intensity profiles of radiation modes in general, but rather are optimized to maximize the power transfer to the fiber, which is often approximated as a Gaussian mode. For high coupling efficiency, the grating coupler should exhibit high directionality toward the optical fiber, thereby minimizing the optical power loss into the substrate. To achieve this, specialized layer structures such as a Bragg or metallic mirror substrate^26–28^ or silicon-overlay structures^29,30^ have been applied, and coupling efficiencies of >60 % have been obtained. Another important factor in the fiber-to-chip grating coupler is the spatial distribution of the radiation mode intensity. A simple uniform-period grating coupler with equal slot widths creates an exponentially decaying radiation pattern in the plane, as the power in the guided mode decreases exponentially. This results in a mismatch between the radiated field and the fiber mode and limits the coupling efficiency^20,21^. Grating apodization can form Gaussian profiles in the radiation mode and has been used to increase the fiber-to-chip coupling efficiency^26,28,31–33^ and to focus the radiating beam at a specified distance^3,34^. By using the apodized gratings, the back-reflection of the guided mode can also be suppressed, and high coupling efficiencies (67–87 %) have been achieved on a common silicon-on-insulator (SOI) platform^26,28,31–33^.

Most grating couplers are designed to interface a photonic mode and an optical fiber mode whose mode field diameter (MFD) is ≈5–10 µm (mode area <10^2^ µm^2^) with the waist located near the surface (<5 μm above). Such small grating couplers are less sensitive to the spatial phase profile of the radiation mode. However, to realize a >100 µm wide free-space beam that can propagate millimeter-scale distances with low diffraction loss, it is essential to accurately control both the phase and intensity profiles of the out-coupled beam. To achieve this, the grating apodization requires careful optimization since varying the duty cycle will change the effective index; the grating pitch should be adjusted accordingly for the beam collimation. In addition, typical grating couplers are designed in 2D cross-sections and the varying mode intensity and phase in the lateral direction, which is parallel to the grating lines, are not considered. The waveguide is simply tapered out so that the waveguide mode expands to the slab mode with a cone shape. While producing acceptable losses for fiber coupling, such an approach fails in beam collimation in the lateral direction, which is important for coupling into spatially extended modes. To shape the radiation beam fully and manipulate the radiation direction in 3D free space, it is essential to design a mode expander that can achieve a specified mode profile and a satisfactory beam collimation. Accurate engineering of the desired radiation mode intensity and phase across the 2D plane is required.

**Fig. 1:**
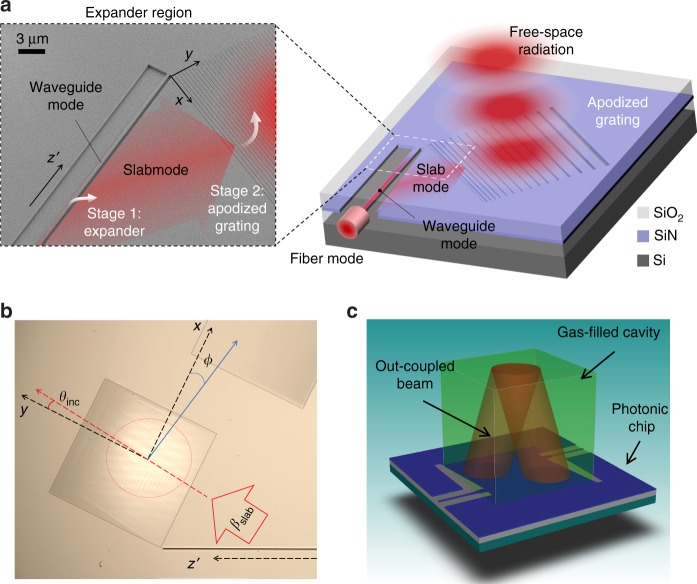
Extreme mode converter. **a** A schematic diagram of the extreme mode converter and an SEM image of the SiN structure without the top SiO_2_ cladding. The red-color overlays illustrate the two-step conversion (stage 1: photonic waveguide mode to 1D-Gaussian slab mode and stage 2: 1D-Gaussian slab mode to 2D-Gaussian beam in free space). The diagram is not to scale. **b** A microscope image of the fabricated extreme mode converter with a coordinate system (*x-* and *y*-axes, incident angle *θ*_inc_, and azimuthal angle *φ*). The propagation direction of the waveguide mode is defined as *z*′. **c** A concept figure of a photonic chip with two extreme mode converters coupling light in and out for optical interrogation (red) of a gas-filled cavity volume (green). A mirror that is placed on top of the gas-filled cavity is used to reflect the beam that is radiated from one grating into the other grating

In this paper, we present an extreme mode converter that can interface with the photonic mode in a waveguide (modal area of ≈300 nm × 250 nm) and the Gaussian beam in a free space (modal area of ≈160^2^ µm^2^). The mode mismatch between the two modes is ≈0.34 × 10^6^ times (in area). The extreme mode converter consists of two stages (as illustrated in Fig. 1a): a waveguide-to-slab mode expander, followed by an apodized grating. First, a 160 µm wide, collimated (one-dimensional) Gaussian slab mode is created. Then, a large apodized grating with straight lines is used to couple it to free space. Separating the two stages and producing a collimated slab mode with a flat wavefront in the first step effectively makes the second stage apodization problem two-dimensional and, therefore, analytically and numerically tractable. Optimizing the spatially varying period and duty cycle of the grating achieves the desired Gaussian intensity and flat wavefront in the orthogonal direction at the 2nd stage. Analytical and numerical methods are combined for the design and optimization. The chip-to-beam conversion is experimentally demonstrated. The mode intensity profile and the wavefront of the generated beams are characterized by capturing the real and Fourier images, respectively. The out-coupling angles (polar and azimuthal) of the beam are quantified and grating-to-grating coupling is achieved, in which the radiating beam is coupled back to the chip by using two extreme mode converters and placing a flat mirror a few millimeters above and parallel to the chip. This configuration is used to quantify experimentally the mode converter efficiency.

## Results

### Modeling: a two-stage extreme mode converter

The extreme mode converter consists of two stages: stage 1 is an expander that converts the photonic waveguide mode into the slab mode and stage 2 is an apodized grating that couples out the slab mode into free space. Figure 1a provides a not-to-scale schematic diagram and a scanning electron microscopy (SEM) image of the chip structure with a color overlay to illustrate the mode transformation. To achieve the extreme mode conversion, the expander in stage 1 is designed to attain a wide 1D-Gaussian intensity profile with a flat wavefront in the slab. The grating in stage 2 is apodized by varying both the grating period and the duty cycle to realize a 2D-Gaussian beam in free space with a large beam waist (*w*_0_ > 100 µm). Specifically, the period is varied such that the phase of the out-coupling beam is designed to be flat to achieve a high-quality beam collimation. Figure 1b shows a microscope image for the fabricated converter, including specified coordinates and angles. *θ*_inc_ is the incident angle between the slab mode *β*_slab_ and the grating vector ${\textstyle{{2\pi } \over \Lambda }}$ (*y*-axis). The azimuthal angle *ϕ* is also defined with respect to the grating lines (*x*-axis). Figure 1c illustrates an application in which a chip with two extreme mode converters is integrated to interrogate a gas-filled cavity in a compact optical system. To demonstrate this concept experimentally, we place a mirror above the chip surface at the plane of the mode overlap from the two converters to characterize the grating-mirror-grating coupling. The devices are designed to operate at the free-space wavelength of *λ*_0 _= 780 nm and silicon nitride (SiN) is chosen as the photonics guiding material to minimize the losses at this wavelength^1^.

#### Stage 1 (expander): waveguide mode to 1D-Gaussian slab mode conversion

**Fig. 2:**
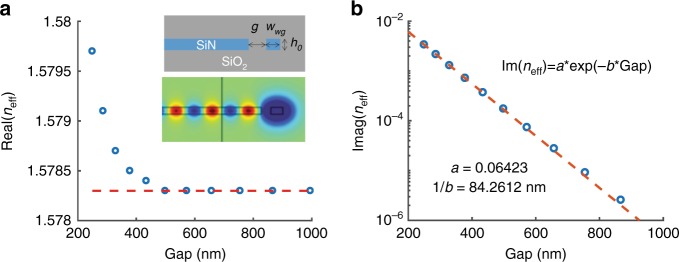
Stage 1: photonic waveguide mode to 1D-Gaussian slab mode conversion via evanescent coupling. The numerically calculated effective refractive index (*n*_eff_) of the photonic waveguide mode as a function of the gap size *g* between the waveguide and the slab: **a** The real part of *n*_eff_ (the dashed orange line: limiting value for *g* *>* 500 nm; inset: cross-sections of the schematic diagram and the FEM domain with the computed TE mode profile) and **b** the imaginary part of *n*_eff_ (the dashed orange line: fitting curve; text: fitting parameters). The height *h*_0_ and the width *w*_wg_ of the waveguide are 250 nm and 300 nm, respectively

Stage 1 is a mode expander that converts the photonic waveguide mode to the slab mode with a flat wavefront and a 1D-Gaussian lateral power density distribution in the *x-*direction (the expander part in Fig. 1a). The basic principle for the mode expander is evanescent coupling. The coupling strength between the waveguide and the slab depends on the gap size *g* between them and the gap profile *g*(*z*′) can be designed to form a Gaussian intensity distribution in the slab (along the waveguide direction of the wave propagation, which is denoted as *z*′). First, to quantify the coupling strength between the waveguide and the slab, a finite element method (FEM) is used to evaluate numerically the complex effective refractive index (*n*_eff_) as a function of *g*. A commercial FEM solver in the frequency domain is used to calculate the waveguide cross-sectional mode profile and its effective index. The power from the waveguide couples to the slab and is radiated in-plane, which results in an imaginary component of the index, thereby accounting for the mode power decay along the expander. To model this within a finite geometrical domain and to avoid the complications of using perfectly matched layers (PMLs) within an eigenmode calculation, we choose to introduce optical losses into the slab material instead. The 1 µm wide part of the slab that is closest to the waveguide is modeled as a perfect (lossless) dielectric and further away from the waveguide the slab material optical loss is increased adiabatically (Supplementary Figure S1). This ensures that there is no reflection of the slab mode back toward the waveguide from either within the slab or the domain boundary (Fig. 2a inset, Supplementary Figure S1, and Supplementary movie 1). The numerical results are independent of the choice of the loss profile if the loss is introduced at least 1 µm away from the gap and increased from 0 gradually over a distance of several micrometers.

Figures 2a and 2b show the real and imaginary parts, respectively, of the simulated *n*_eff_. The insets in Fig. 2a show a schematic diagram of the simulation domain and the mode profile of the fundamental transverse-electric (TE_0_) mode. All the devices in this paper are designed based on the TE_0_ mode. The thickness of the SiN layer (waveguide and slab), which is denoted as *h*_0_, is set to 250 nm and the waveguide width, which is denoted as *w*_wg_, is set to 300 nm. All elements are made of SiN and clad with SiO_2_ (upper: >1 µm, lower: ≈2.9 µm). The refractive indices of SiN and SiO_2_ are 2.01 and 1.45, respectively, at *λ*_0_=780 nm.

Re(*n*_eff_) corresponds to the propagation constant *β* *=* Re(*n*_eff_)*β*_0_ of the waveguide mode (*β*_0_ = 2*π/λ*_0_ is the free-space wavenumber) and determines the tilt-angle *θ*_tilt_ of the slab-mode direction of propagation relative to the waveguide. For the chosen value of *h*_0_, the effective index of the 1D TE slab mode is calculated to be 1.79 and the tilt angle between the waveguide and slab modes can be estimated by *θ*_tilt = _cos^−1^(Re(*n*_eff_)*/*1.79). In Fig. 2a, Re(*n*_eff_) approaches 1.578 for *g* *>* 500 nm. This indicates that the evanescent-coupled slab mode would have the same tilt angle, namely, *θ*_tilt_ = cos^−1^(1.578*/*1.79) = 28.18°, for gap sizes that exceed 500 nm. In other words, the evanescent-coupled waves in the slab will be collimated if *g*(*z*′) > 500 nm. In Fig. 2b, Im(*n*_eff_) corresponds to the power loss of the evanescent coupling and decreases approximately exponentially as the gap size increases. Using this data and assuming adiabatic variation of the gap profile, *g*(*z*′) can be designed for the desired power distribution along *z*′. The phase at the slab boundary is the same as the phase in the waveguide and increases linearly along *z*′ for a constant value of Re(*n*_eff_). For *g*(*z*′) > 500 nm, the variation in gap size does not shift *θ*_tilt_ and only affects the power distribution. Smaller gaps can be employed; however, variation in Re(*n*_eff_) may need to be compensated. The slab boundary and the waveguide can be appropriately curved to achieve the desired wavefront for the slab mode. In our design, the slab edge is straight, thereby creating a flat wavefront for the collimated slab mode.

The following procedures are used to design *g*(*z*′) and achieve the correct Gaussian intensity profile of the slab mode. The optical power in the waveguide, which is denoted as *P*(*z*′), can be expressed as

1 $$\frac{{dP\left( {z\prime } \right)}}{{dz\prime }} = - P\left( {z\prime } \right)\alpha \left( {z\prime } \right)$$

where *α*(*z*′) is the loss coefficient, which can be expressed as $\alpha \left( {z\prime } \right) = \frac{{4\pi }}{{\lambda _0}}{\rm{Im}}\left( {n_{{\rm{eff}}}} \right)$. The initial power in the waveguide is *P*(−∞) = 1. The power density in the slab $\frac{{dP_{\rm s}\left( {z\prime } \right)}}{{dz\prime }}$ forms a Gaussian mode with a beam waist of *w* and can be represented as:

2 $$\frac{{dP_{\rm s}\left( {z\prime } \right)}}{{dz\prime }} = C\exp\left( { - \frac{{2z\prime ^2}}{{w^2}}} \right)$$

The coefficient, which is expressed as $C = \frac{1}{w}\sqrt {\frac{2}{\pi }}$, is obtained by setting the total power to 1 and integrating: ${\int}_{-\infty }^{\infty} {\frac{{dP_{\rm s}\left( {z\prime } \right)}}{{dz\prime }}dz\prime = 1.}$ Per energy conservation, the total power in the waveguide and the slab should be equal to 1, i.e., *P*(*z*′) + *P*_*s*_(*z*′) = 1, and the loss in the waveguide should be equal to the coupling power of the slab at that segment, i.e., *dP*_s_(*z*′) = −*dP*(*z*′). Rewriting these two conditions, the following equations are obtained:

3 $$P(z\prime ) = 1 - {\int}_{-\infty}^{z\prime} {\frac{1}{w}\sqrt {\frac{2}{\pi }} \exp \left( {\frac{{ - 2\zeta ^2}}{{w^2}}} \right)d\zeta }$$

4 $$\frac{1}{w}\sqrt {\frac{2}{\pi }} \exp \left( {\frac{{ - 2z^{\prime 2}}}{{w^2}}} \right) = P\left( {z\prime } \right)\frac{{4\pi }}{{\lambda _0}}\rm{Im} \left( {n_{{\rm{eff}}}} \right)$$

Solving these two equations and using the relation ${\rm{Im}}\left( {n_{{\rm{eff}}}} \right) = a{\exp} \left( -bg(z\prime) \right),$ where *a* and *b* are the fitting coefficients from Fig. 2b, the gap profile *g*(*z*′) is:

5 $$g(z\prime ) = \frac{1}{b}\ln \left\{ {\frac{1}{a}\frac{{\lambda _0}}{{\sqrt 2 \pi ^{3/2}w}}\frac{{\exp \left( { - 2z\prime /w^2} \right)}}{{1 - {\rm{erf}}\left( {\sqrt 2 z\prime /w} \right)}}} \right\}$$

The beam waist *w* is of the Gaussian distribution along the waveguide direction. The actual beam waist *w*_0_ of the resulting 1D-Gaussian slab mode, which is normal to its direction of propagation in the slab, is obtained as *w*_0_ = *w* sin(*θ*_tilt_). In our design, the beam waist is set to *w*_0_ = $100\sqrt 2$ µm.

#### Stage 2: 1D Gaussian slab mode to 2D Gaussian beam conversion

Stage 2 is an optimized apodized grating with a spatially varying duty cycle and period that outcouples the 1D Gaussian slab mode into the 2D free-space Gaussian. The grating lines are straight and parallel. The slab mode is collimated so that the phase is invariant along the grating lines, while the intensity is varied only gradually. Therefore, to create the Stage 2 out-coupler, it is sufficient to solve a 2D problem with translational invariance along the grating lines to create a collimated Gaussian profile in the plane that is normal to the grating lines (in the *y-*direction).

For a collimated Gaussian output, further simplifications could have been applied by leveraging the slow variation of the intensity and phase across the grating. However, the 2D TE scattering problem from the slab mode into the free space can be quickly and accurately solved for the ≈300 µm grating using a commercial finite element frequency domain solver. This makes it possible to apply a more general numerical optimization technique to solve the inverse problem of finding a grating design that optimizes the coupling between the input slab mode and any arbitrary prescribed free-space mode (i.e., the “inverse design”). By adding a geometric deformation field into the finite element problem setup, we could employ an efficient gradient-based optimization technique to optimize the values of the 14 scalar parameters that define the device geometry and the Gaussian beam, including the spatially varying period and duty cycle, as described in the Materials and Methods section.

**Fig. 3:**
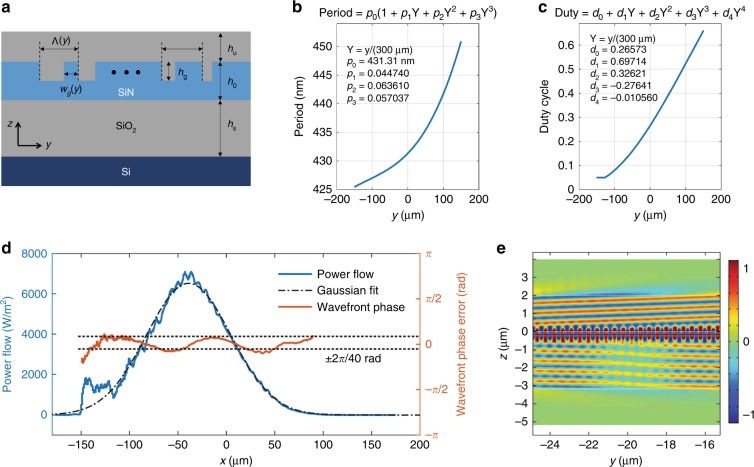
Stage 2: 1D-Gaussian slab mode to 2D-Gaussian beam conversion. **a** A schematic diagram of the apodized grating with the following geometric parameters: *h*_0_ = 250 nm, *h*_g_ = 85 nm, *h*_s_ = 2.9 µm, and *h*_u_ = 2.8 µm. The grating period Λ(*y*) and grating width *w*_*g*_(*y*) are apodized. The numerically optimized grating **b** period Λ(*y*) and **c** duty cycle 1-*w*_*g*_(*y*)*/*Λ(*y*) (insets: optimized polynomial coefficients). **d** FEM results of the out-coupled beam: power flow (blue), Gaussian fit (black dash-dot line), and wavefront phase error (orange). Dashed lines indicate the upper and lower bounds of the phase error, which are within ± 2*π/*40. **e** The electric field profile (*E*_*x*_) within a portion of the FEM simulation domain

Figure 3a shows a schematic diagram of the resulting apodized grating in the *yz*-plane with spatially varying period Λ(*y*) and grating width *w*_*g*_(*y*). The duty cycle is defined as 1-*w*_*g*_(*y*)*/*Λ(*y*). The thicknesses of the layers are *h*_0_ = 250 nm, *h*_g_ = 85 nm, *h*_s_ = 2.9 µm, and *h*_u_ = 2.8 µm. Figures 3b and 3c show the optimization results for the grating period and duty cycle, respectively. The insets in each figure show the optimized polynomial coefficients. Figure 3d shows the numerically simulated out-coupling power flow (blue), its Gaussian fit (black dash-dot line), and the wavefront phase error (orange) when the geometric parameters of Figs. 3b and 3c are used. Figure 3e is the magnified view of the simulated electric field profile (*E*_*x*_). The resulting optimal out-coupling angle of the Gaussian is 2.2° in free space and the wavefront error is <2*π/*20 rad over the beam. The power distribution fits well with the Gaussian profile; however, the beam full width at half maximum (FWHM) is ≈103 µm, which is ≈15% lower than the desired outcome. The port width is numerically forced to FWHM = 117 µm by an added term in the cost function and a constraint and a small coupling penalty is associated with this width mismatch. We speculate that the optimization algorithm is balancing this mismatch loss with additional losses (increased wavefront error and loss into the substrate) that are associated with extending the Gaussian or, alternatively, that the parameters of the optimization algorithm are not selected perfectly, thereby resulting in a small residual error. The calculated Gaussian port coupling is 68 %; the power that is flowing down into the substrate is 26 %; the slab mode reflection and transmission are negligible. The remaining power accounts for the mode mismatch with the Gaussian port.

Due to the constructive/destructive interference with the downward-outcoupled light that is back-reflected up from the Si wafer surface, the numerical simulation demonstrates a periodically varying upward coupling efficiency from ≈67 % for the optimum oxide thickness down to ≈35 % for ≈150 nm thicker or thinner oxide. As the destructive interference decreases the out-coupled intensity, the optical power in the slab mode propagates further along the grating in the *y-*direction, thereby resulting in significant widening of the out-coupled Gaussian beam. Experimentally, it is likely that some oxide thickness variation existed between runs, which contributed to the observed Gaussian beam width variation in the *y-*direction.

### Experiment 1: Gaussian beam characterization

To demonstrate the extreme mode converter that was designed in Section 2, we have fabricated and tested the devices, as described in the Materials and Methods section.

#### Free-space mode intensity profile

**Fig. 4:**
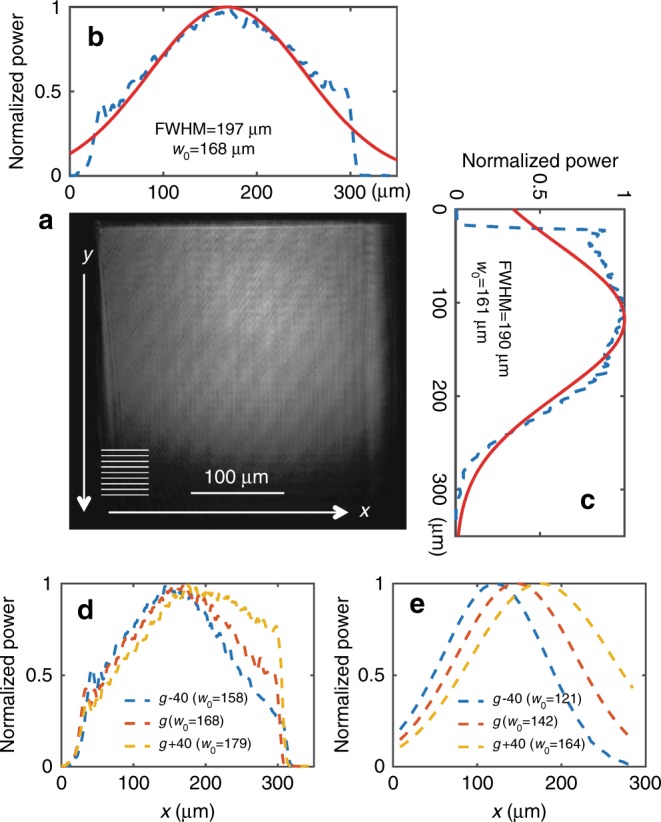
Gaussian mode profile on a grating. **a** A microscope image of a converted Gaussian beam on a 300 µm×300 µm grating. The grating lines are parallel to the *x*-axis (as schematically indicated). The scale is calibrated based on the known physical size of the grating. **b**, **c** are the projected images of **a**, which show the Gaussian mode profiles along the *x-* and *y-*axes, respectively (blue dashed lines: data; red solid lines: fitting curves). The full width at half maximum (FWHM) and the beam waist *w*_0_ are shown in each figure. **d** Measured mode profiles (projected on *x*-axis) for various gap sizes that have constant ± 40 nm variations on the gap profile *g*(*z*′) (blue: *g* - 40 nm, orange: *g*, and yellow: *g* *+* 40 nm). **e** Numerically calculated (ODE) mode profiles that are similar to **d**. The uncertainties in the characterized beam waist are approximately ± 1 µm, as determined by the Gaussian fit

To characterize the mode profiles of the converted Gaussian beam, microscope images of the beam on the grating were captured. Figure 4a shows a microscope image of the converted Gaussian beam. The inset shows the direction of the gratings: the grating lines are parallel to the *x*-axis and perpendicular to the *y*-axis. The slab mode is incident from the top of the image. Figures 4b and 4c show the normalized powers of the beam that are integrated along the *y-* and *x-*directions and projected to the *x-* and *y-*axes, respectively. The dashed blue lines are the data and the red lines are the reference Gaussian curves. The FWHM and the beam waist, namely, *w*_0_, of each projection are also shown in each figure. The power distributions of the beam fit well with the Gaussian curves within the grating area and the beam waist on both the *x-* and *y-*axes is *w*_0_ ≈160 µm, which is reasonably close to the design target value (*w*_0 _$= 100\sqrt 2$ = 141.4 µm). The fabrication imperfections and the index differences between the model and the real materials may have caused these errors. In Fig. 4c, the Gaussian shape is cut at the beginning part of the gratings; this is due to the minimum feature size limit of the grating (20 nm) in the fabrication, which is similar to the FEM result in Fig. 3d.

Uniformly increasing or decreasing *g*(*z*′) shifts the center position of the slab mode and the Gaussian mode in the grating (along the *x-*axis). It also affects *w*_0_ and the FWHM in the *x-*direction. Figures 4d and 4e show the measured and numerically calculated intensity mode profiles that are projected to the *x*-axis. For the numerical calculation, Eq. 1 is solved via the ordinary differential equation (ODE) solver with the gap profile *g*(*z*′) of Eq. 5. The orange line is the original design with the *g*(*z*′) and the blue and yellow lines are cases in which the gap is uniformly decreased or increased, respectively, by 40 nm, i.e., *g*(*z*′)−40 nm and *g*(*z*′)+40 nm. In both the experimental and numerical cases, the narrower gap size shifts the center position to the left (closer to the beginning of the evanescent coupler). A narrower gap increases the coupling, thereby increasing the slab mode intensity at the beginning of the coupler; hence, less light remains in the waveguide to couple out toward the end, thereby decreasing the slab mode intensity there. The opposite is observed for a wider gap. Furthermore, the smaller gap size yields a narrower *w*_0_; again, opposite is observed for the larger gap size. In the numerical results, *g*(*z*′)+40 nm yields *w*_0_ = 164.0 µm, which is similar to the beam waist from the experiment (*w*_0_ ≈168 µm ± 1 µm); this gap variation is a possible reason for the ≈15 % width difference between the designed *w*_0_ = 141.4 µm and the experiments.

#### Wavefront

**Fig. 5:**
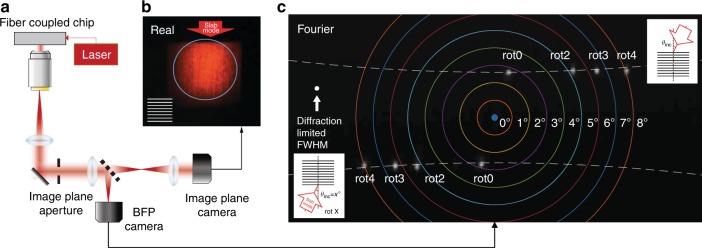
Back focal plane (BFP) measurement. **a** The BFP measurement setup for characterizing the beam profile in both angular (Fourier) space and real space. **b** Real-space image of the converted Gaussian beam (blue line: image plane aperture with a diameter of 250 µm). **c** A BFP composite image of the Gaussian beams for various grating rotational angles relative to the incident slab mode (rot0: 90° − *θ*_tilt_ = 61.82° rotation from the expander waveguide, rot2 = rot0 + 2°, rot3 = rot0+3°, and rot4 = rot0+4°). The BFP image X (Y)-axes are oriented along (normal to) the grating lines in **b**. Two identical devices that are rotated 180° are measured to establish the origin (surface-normal). The white dashed lines are the theoretical out-coupling angles that are obtained via Eqs. 7 and 8. A white dot indicates the ideal diffraction-limited beam’s full width at half maximum (FWHM)

To check the beam collimation of the converted Gaussian mode, its far-field intensity is measured as a function of the angle (Fourier space) by capturing the back focal-plane (BFP) images (Fig. 5a; Materials and Methods). A movable variable-diameter circular aperture was placed in the image plane of the microscope, before the beam splitter, thereby enabling full or spatially selective evaluation of the far-field light, i.e., of light that is coming from the whole grating or any part of the grating that is selected by the aperture. Figure 5b shows a real image at the grating and the blue line indicates the outline of the aperture with a diameter of ≈250 µm. Figure 5c shows the angular (Fourier) space images at the BFP; the colored lines represent the grid (one degree/line) of the polar angle *θ*. The gray spots are the actual beam images from each device. All spots are of small size and near diffraction-limited; otherwise, the spots would spread broadly over wider angles. The white dot serves as a guide for the eye, with a diameter that corresponds to the ideal diffraction-limited beam’s FWHM. For each device set, we have two identical mode converters with opposite orientations (mode converter 1 is rotated 180° relative to mode converter 2); thus, in Fig. 5c, the upper four gratings are in opposite orientations relative to the lower four gratings. Device rot0 is a device whose grating lines are nominally orthogonal to the incident slab mode, which is designed to have a tilt angle of *θ*_tilt_ = 28.18° relative to the waveguide. Devices rot2, rot3, and rot4 are devices with additional rotations of 2°, 3°, and 4°, respectively, of the grating relative to the incident slab mode. We have rotated the gratings to engineer the Gaussian beam polar angle and direction. One aim is to maximize the modal overlap between the two beams at a certain height such that a flat mirror can be used to couple a beam from one device into the other, as illustrated in Fig. 1c. A detailed analysis follows in Section 4.

**Fig. 6:**
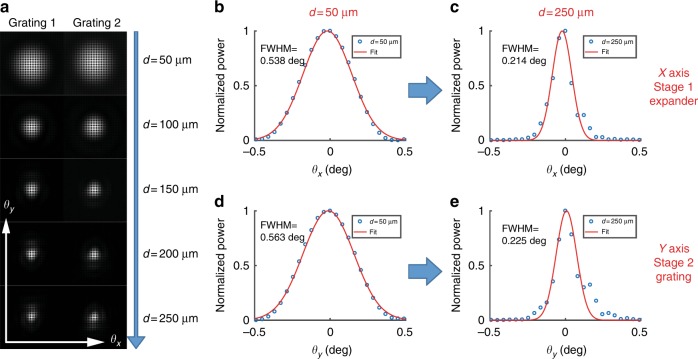
Near-diffraction-limited Gaussian beam. **a** Magnified back-focal-plane (BFP) images from mode converters 1 and 2 for various aperture diameters from 50–250 µm. Clipping the beam by the aperture increases diffraction. **b**–**e** Normalized powers of the BFP images of (**a**) at the center of each axis: (**b**, **c**) along *θ*_*x*_ and (**d**, **e**) along *θ*_*y*_ (blue circles: data; red lines: fitting Gaussian curves). (**b) and **(**d)** are cases in which the aperture diameter is *d* ≈ 50 µm, while (**c**) and (**e**) are cases in which *d* ≈ 250 µm. The uncertainty in the characterized FWHM is approximately ±0.005°, as determined by the Gaussian fit

Figure 6a shows a magnified view of the beam spots (rot0) from top to bottom with image-plane aperture diameters of *d* ≈ 50 µm to 250 µm (left: grating 1, right: grating 2). As the aperture size increases in real space, the spot size in the Fourier domain decreases accordingly. Figures 6b–6e show cross-sections of the normalized powers through the center of the spots (blue dots: data, red lines: fitting curves). Figures 6b and 6c show the cross-sections along *θ*_*x*_ for *d* ≈ 50 µm and *d* ≈ 250 µm, respectively, and Figs. 6d and 6e show the cross-sections along *θ*_*y*_ for *d* ≈ 50 µm and *d* ≈ 250 µm, respectively. The Gaussian distribution in the *x-*direction (or *θ*_*x*_) relates to the mode expansion from the waveguide to a slab mode (stage 1), while the Gaussian distribution in the *y* direction (or *θ*_*y*_) is formed by the apodized grating during stage 2; they are formed by the two independent stages. More importantly, for a large aperture of *d* ≈ 250 µm, as in Figs. 6c and 6e, the measured angular FWHMs are close to the expected FWHM of a diffraction-limited beam, namely, FWHM=$\frac{{2\sqrt {2{\ln}2} \lambda _0}}{{w_0\pi }} = 0.2094^ \circ$ for *w*_0_=160 μm, and demonstrate a good beam collimation.

### Experiment 2: out-coupling angles, mode converter efficiency, and grating-to-grating coupling

To integrate and optically couple the photonic chips with other systems in free space, the extreme mode converter can be used as a building block for establishing optical coupling between the systems. To explore such an engineering opportunity, we have placed two couplers with opposite orientations on the same photonic chip and arranged their locations and outcoupling angles (polar and azimuthal) to create a large beam overlap several millimeters above the chip. Finally, a flat mirror is introduced at that location and grating-to-grating coupling experiments are conducted to quantify the coupling of the generated beam back to the photonic system. Using this setup, we can also evaluate experimentally the overall mode converter efficiency and account for the mode mismatch loss between identical mode converters.

**Fig. 7:**
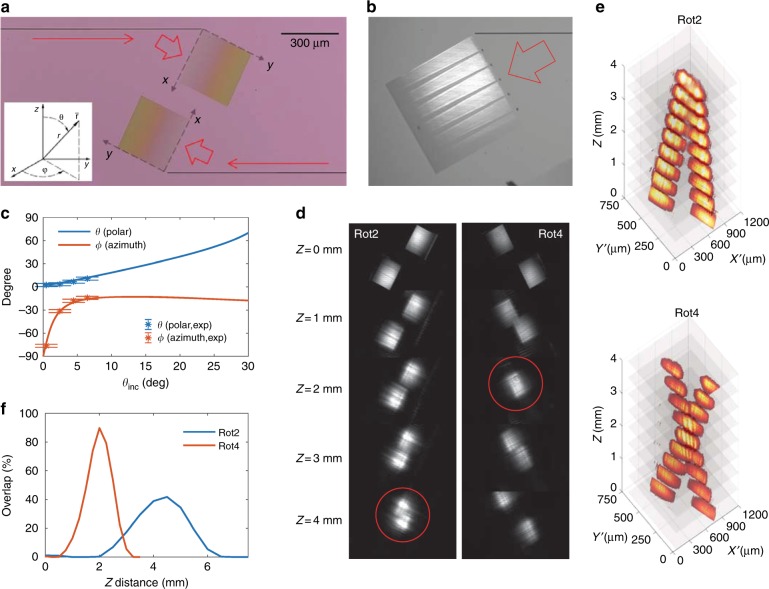
Out-coupling angles and modal overlap. **a** A microscope image of the two extreme mode converters. The inset shows the definitions of the polar (*θ*) and azimuthal (*φ*) angles. **b** A magnified converter image with scatterers that are embedded into the slab. **c** Polar (*θ*, blue) and azimuthal (*φ*, orange) angles of the out-coupled beam as functions of incident angle *θ*_inc_. The solid lines are analytical calculations and the points with error bars are the measured angles. The error bars indicate the one-standard-deviation uncertainties that are propagated from the beam center estimates in the images. **d** Microscope images of the two out-coupling beams at various distances *z* (left: rot2; right: rot4). The red circles indicate the *z*-positions of the maximum overlap. **e** 3D stack images of **d**. **f** Measured modal overlap percentages of the two out-coupling beams as a function of *z* (blue: rot2 and orange: rot4)

Figure 7a shows a microscope image of the two extreme mode converters facing in opposite directions with a center-to-center separation distance of ≈475 µm. In Fig. 1b, the incident angle *θ*_inc_ is defined for the angle between the slab mode *β*_slab_ and the grating vector Λ (*y-*axis). For devices rot0, rot2, rot3, and rot4, the designed θ_inc_=0°, 2°, 3°, and 4°, respectively. The tilt angle *θ*_tilt_ is fixed at the nominal value of 28.18° and the gratings are rotated to adjust *θ*_inc_. In addition, the *x-* and *y*-axes are referenced to the grating, not a global frame. The azimuthal angle *φ* and the polar angle *θ* are defined with respect to the grating lines (*x-*axis) and the surface normal (*z-*axis), respectively (inset of Fig. 7a). To verify the propagation direction of the slab mode (*θ*_tilt_) after stage 1 (expander), in a separate experiment, a nominally identical device was co-fabricated, in which a series of holes in front of the grating were deliberately introduced to serve as scatterers for the slab mode. Figure 7b shows a microscope image of such a device (rot0) with TE_0_ input. The scatterers form shadows in the slab mode along its propagation direction, which are made visible by the grating. As expected, the angles of the shadows are close to *θ*_inc_ ≈ 0^◦^ and the long, uniform-contrast shadows qualitatively demonstrate satisfactory collimation of the slab mode. This further validates the performance of the mode expander.

The polar *θ* and azimuthal *φ* angles of the out-coupling beam can be engineered by varying the incident angle *θ*_inc_ via grating rotation. Due to the momentum conservation in the grating plane, the out-coupling beam angles should follow

6{\rm a} $$k\sin \theta \sin \phi = \beta _y - \frac{{2\pi m}}{{\rm{\Lambda }}}\quad \left( {m} {\rm : integer} \right)$$

6{\rm b} $$k\sin \theta \cos \phi = \beta _y$$

where *k* *=* 2*π/λ*, *β*_*y*_ = *β*_slab_ cos(*θ*_inc_), *β*_*x*_ = *β*_slab_ sin(*θ*_inc_), Λ is the effective grating period, and the propagation constant of the slab mode can be represented as $\beta _{{\rm{slab}}} = \frac{{2{\rm{\pi }}}}{{\lambda _0}}n_{{\rm{eff}}}$. Rewriting Eq. 6 for *θ* and *φ*,

7 $$\sin \theta = \sqrt {\left( {n_{{\rm{eff}}}\sin \theta _{{\rm{inc}}}} \right)^2 + \left( {n_{{\rm{eff}}}\cos \theta _{{\rm{inc}}} - \frac{{m\lambda_0 }}{\Lambda }} \right)^2}$$

8 $$\tan \phi = \frac{{\cos \theta _{{\rm{inc}}} - \frac{{m\lambda _0}}{{n_{{\rm{eff}}}\Lambda }}}}{{\sin \theta _{{\rm{inc}}}}}$$

To avoid losing light into multiple diffraction orders and to create a near-vertical Gaussian beam for *θ*_inc  _= 0, *m* *=* 1 is chosen and $1 \gg n_{{\rm{eff}}}\left( {\frac{{\lambda _0}}{{\Lambda n_{{\rm{eff}}}}} - 1} \right) > \,0$. The blue and orange lines in Fig. 7c show the polar (*θ*) and azimuthal (*φ*) angles as functions of *θ*_inc_, which follow Eqs. 7 and 8, respectively. For the parameters, *n*_eff _=  1.79 and Λ = 425 nm are assumed. The points represent the experimentally measured angles for *θ*_inc_=0°, 2°, 4°, and 6° and match the analytical estimates. Figure 7d shows the captured images of the rot2 (left) and rot4 (right) devices from this set at various *z-*positions. For each device, the *z-*positions of the maximum modal overlap are marked with red circles (≈4 mm for rot2 and ≈2 mm for rot6). Figure 7e shows the 3D stack images of Fig. 7d, the azimuthal angles and the mode overlap positions.

We have also experimentally estimated the overlap percentage of the two beams from each converter. Each of the beam images was captured at various heights and the following equation was used to extract the modal overlap:

9 $${\rm{Overlap}}\left( z \right) = \frac{{{\int} {\sqrt {I_{1}\left(z\right)I_{2} \left(z\right)} dA} }}{{\sqrt {{\int} {I_{1} \left(z\right)dA} {\int} {I_{2} \left(z\right)dA} } }} \times 100\,\,{{\% }}$$

where *I*_1_(*z*) and *I*_2_(*z*) are the beam intensity images from each device, captured separately. The intensity measurement is not phase sensitive and this equation provides an upper bound on the expected mode coupling loss by assuming perfect phase matching between the two beams, such as perfect collimation with beam waists at the mirror location. The blue line in Fig. 7f represents the measured overlap percentage of rot2 and the orange line in Fig. 7f represents that of rot4. As expected from Fig. 7f, the *z-*positions of the maximum overlap for rot2 and rot4 are ≈4.5 mm and ≈2 mm, respectively. The maximum overlap percentage for rot4 exceeds 90 %, thereby fulfilling a necessary condition for low-loss grating-to-grating coupling, in which the radiating beam that is produced by one mode converter is reflected back to the chip (and a second converter) by a mirror that is placed at this position.

**Fig. 8:**
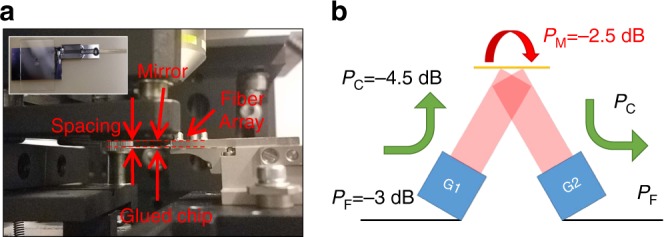
Grating-to-grating coupling. **a** The measurement setup of the grating-to-grating coupling. The inset shows the top view of the chip, which is glued with a fiber array. **b** A schematic diagram of the loss components from each section

Figure 8a shows the experimental setup for the grating-to-grating coupling that is used to characterize the single-mode coupling efficiency of the mode converter in a realistic application scenario. The photonic chip is glued to a fiber array for input/output coupling and a mirror is placed ≈2 mm above the chip, thereby resulting in maximum overlap for this rot4 device. The inset image shows the top view of the chip with the glued fiber array. Figure 8b shows a schematic diagram of the entire device structure with key loss parameters: *P*_F_: the fiber-to-chip edge-coupling loss; *P*_C_: the connecting waveguide and extreme mode converter loss; and *P*_M_: the mode-mismatch loss between the two beams. The typical loss for the edge-coupling is *P*_F_ = −3 dB ± 0.5 dB, which was determined via multiple measurements using short waveguide loop-back structures that were connected to the inverse-tapered couplers. Here and below, the uncertainties are measured and the statistical uncertainties are propagated one standard deviation. Measuring the outgoing Gaussian beam power and assuming the abovementioned value for the edge-coupling, the loss for the extreme mode converter, including the SiN waveguide, stage 1 mode expander and stage 2 grating coupler, is *P*_C_ = −4.5 dB ± 0.5 dB, which corresponds to an overall experimental fiber to Gaussian beam loss of approximately *P*_F_ + *P*_C_ =  −7.5 dB ± 0.5 dB for each device. Using the setup in Fig. 8a, the light is successfully coupled back to the chip. By subtracting the independently measured fiber-to-Gaussian-beam losses for each of the two mode converters from the total fiber-to-fiber loss, the excess loss due to the mode-mismatch between the two beams is experimentally determined to be *P*_M_ = −2.5 dB ± 1.0 dB, which results from a combination of lateral and angular beam misalignments and wavefront errors.

## Discussion

An extreme mode converter that can efficiently couple a few-hundred-nanometer-wide photonic waveguide mode to a hundred-micrometer-wide free-space Gaussian beam has been designed, numerically optimized, and experimentally demonstrated. This expansion corresponds to an increase in the mode area by a factor of 0.34 × 10^6^. General guidelines for designing such a mode converter are presented and this approach can be applied to other types of mode conversions as well. Specifically, the evanescent expander offers a novel, optically broadband approach for coupling single-mode waveguides to wide slab modes with arbitrary profiles. We have successfully demonstrated the mode conversion experimentally and generated a Gaussian beam with a beam waist of *w*_0_ ≈160 µm. The converted Gaussian beam is well-collimated and approaches the diffraction limit, as confirmed by the BFP measurements. Furthermore, the ability to engineer the out-coupling beam direction is presented and used to demonstrate a low-loss grating-to-grating coupling between two converters on the same chip, with ≈4 mm free-space propagation distance. This extreme mode converter can be used as a building block for the interaction of the photonic chip with other systems, thereby enabling novel applications in atomic physics, biological and/or chemical sensing, LIDAR, and biomedical health-care systems, among others.

## Materials and methods

### Stage 2: large apodized grating design via gradient-based numerical optimization

We apply a general numerical optimization technique to solve the inverse problem of finding a grating design that optimizes the coupling between the input slab mode and any arbitrary prescribed free-space mode (i.e., “inverse design”). Optimization of smaller plasmonic and protonic grating couplers has been achieved^40–42^ via gradient-based optimization methods^43^. Although the cost function gradients are calculated efficiently, the previous work is limited to either discrete changes in the design geometry or requires the finite element model to be remeshed. Here, a common commercial frequency-domain finite element solver package is used. Following^44^, in addition to the electromagnetic (EM) fields, the spatially dependent deformation vector field (*u,v*), which is discretized on the same mesh, is introduced. This field encodes a continuous shift for each mesh node^45^ and enables continuous and smooth model deformation, together with the mesh, thereby avoiding the digital noise and calculation overhead that are associated with discrete rebuilding and remeshing of the grating model. This is the key to efficient numerical optimization because not only can the EM fields be numerically computed for a particular deformation, but also the gradients of any EM-dependent cost function with respect to all geometrical parameters can be computed cost-effectively^43^. This enables the application of efficient gradient-based nonlinear optimization methods, such as sparse nonlinear optimizer (SNOPT)^46^, which are already implemented in the optimization add-on to the same commercial package^44,47^.

The grating geometry is defined using 11 scalar variable parameters. Two parameters define the grating etch depth and the thickness of the SiO_2_ layer that separates the SiN grating from the Si wafer. The spatially dependent grating duty cycle is described by a 4th-order polynomial function of the location *y* (Fig. 3c), while the grating period is specified by a 3rd-order polynomial of *y* (Fig. 3b). Polynomial coefficients represent the other 9 variables that define the geometry. The duty cycle is additionally constrained from decreasing below 0.05 (≈20 nm) to account for the nanofabrication limits on producing extremely narrow grating lines. Qualitatively, the spatially varying duty cycle, together with the grating depth, controls the strength of the local optical coupling between the slab mode and free space. The spatially varying period ensures that the Gaussian wavefront is planar by compensating for the duty-cycle-dependent effective index of the slab mode. The varying oxide depth ensures that the reflection from the Si wafer constructively interferes, thereby maximizing the optical power in the upward direction.

While it is possible to apply the prescribed deformations only to the model geometric boundaries and obtain a smooth mesh deformation by solving for the “numerically induced” deformation at the internal mesh points^44,45^, we have further reduced the computational complexity by explicitly defining all deformations everywhere in the model as linear interpolations between the prescribed vertical and horizontal displacements of the grating boundaries. In other words, the deformation fields everywhere are explicit functions of the location within the model and the 11 deformation variables, thereby creating the desired model deformations that are described by the two polynomials and two thickness parameters.

The algorithm maximizes the squared modulus of the S-parameter (scattering matrix element), which describes the optical coupling between the slab mode input port and a Gaussian mode output port that is defined on the stationary horizontal domain boundary in free space above the grating (backed up by a PML to eliminate reflection). The vertical domain size is chosen to be large enough for the evanescent fields from the grating to decay before reaching the boundary. The 2D (cylindrical) Gaussian mode waist center was constrained to the grating surface, but allowed to shift in the *y-*direction along the grating. The Gaussian waist center location, the width and the angle are used as 3 additional variable optimization parameters. Allowing the Gaussian waist width to vary prevents the optimization algorithm from becoming stuck in the local optima that arise when the angularly narrow, spatially wide Gaussian port matches a sharp side-lobe of the extended grating out-coupling pattern. In contrast with gradient-free methods, within the gradient-based optimization using the deformed geometry, adding extra variables does not drastically increase the computation time. The Gaussian width was forced to *w*_0_ = 100 µm by adding a term to the optimization cost function that maximizes the waist, while the waist was constrained from above by 100 µm. [*w*_0_ = 100 µm is the prescribed field waist and $E \sim {\exp}\left( { - x^2/{w}_0^2} \right)$; the corresponding FWHM is $w_0\sqrt {2{\ln}2}$ = 117 µm].

### Device microfabrication

The fabrication starts with a 100 mm diameter silicon wafer on which thermal oxide is grown. The design target thickness is 2.9 µm; however, experimentally, this may have varied by 100 nm or more between fabrication runs, which contributes to run-to-run variability in device performance. In the following step, an ≈250 nm thick nominally stoichiometric silicon nitride (SiN) layer is deposited via low-pressure chemical vapor deposition (LPCVD) and patterned twice. First, electron beam lithography is used to define ≈300 nm wide waveguides, inverse-tapers for coupling to the optical fiber and the Stage 1 expander, which is comprised of a variable gap between the waveguide and a slab. The nitride is patterned by a reactive ion etch all the way through the layer. In the second electron beam patterning step, the apodized gratings of Stage 2 are defined and the grating groves are etched nominally 85 nm deep into the nitride layer. A 2.8 µm thick layer of silicon dioxide is deposited via plasma-enhanced chemical vapor deposition (PECVD), after which the wafers are diced and the edges of the chips are polished to expose the ends of the inverse-tapered waveguide fiber couplers. Device layouts were created with the NIST Nanolithography Toolbox^48^.

### Device characterization

For the device characterization, monochromatic laser light (*λ*_0_ ≈780 nm) is coupled from an optical fiber to the waveguide mode (TE_0_) through a tapered fiber-to-chip edge coupler. The input fiber arrays could be permanently attached to the mode converter chip using ultraviolet light curable epoxy. The extreme mode converter transforms the TE_0_ mode to the free-space Gaussian beam and the mode intensity profile and the wavefront of the out-coupled Gaussian beam were characterized by measuring the microscope images in the real and Fourier spaces, respectively. Figure 5a shows the measurement setup for simultaneous Fourier- and real-space imaging. Two charge-coupled device (CCD) cameras were placed at the real and BFP image planes to capture the real- and Fourier-space images, respectively.

## Electronic supplementary material

Supplemental Information

Supplementary movie 1
