# Supplementary material for: Photonic waveguide to free-space Gaussian beam extreme mode converter
Source: Light Sci Appl. 2018 Oct 10;7:72. doi: 10.1038/s41377-018-0073-2 (PMC6177431; doi:10.1038/s41377-018-0073-2)
Supplement: Supplementary file 1 — Supplemental Information [file 41377_2018_73_MOESM1_ESM.docx]

**Supplementary Information:
“Photonic waveguide to free-space Gaussian beam
extreme mode converter”**

Sangsik Kim,^1,2,3^ Daron A. Westly,^1^ Brian J. Roxworthy,^1^ Qing Li,^1,2^

Alexander Yulaev,^1,2^ Kartik Srinivasan,^1^ and Vladimir A. Aksyuk^1, ∗^

^1^*Center for Nanoscale Science and Technology, National Institute of Standards and Technology, Gaithersburg, Maryland 20899, USA*

*^2^ Maryland Nanocenter, University of Maryland, College Park, MD 20742 USA*

*^3^ Department of Electrical and Computer Engineering, Texas Tech University, Lubbock, TX 79409, USA.*


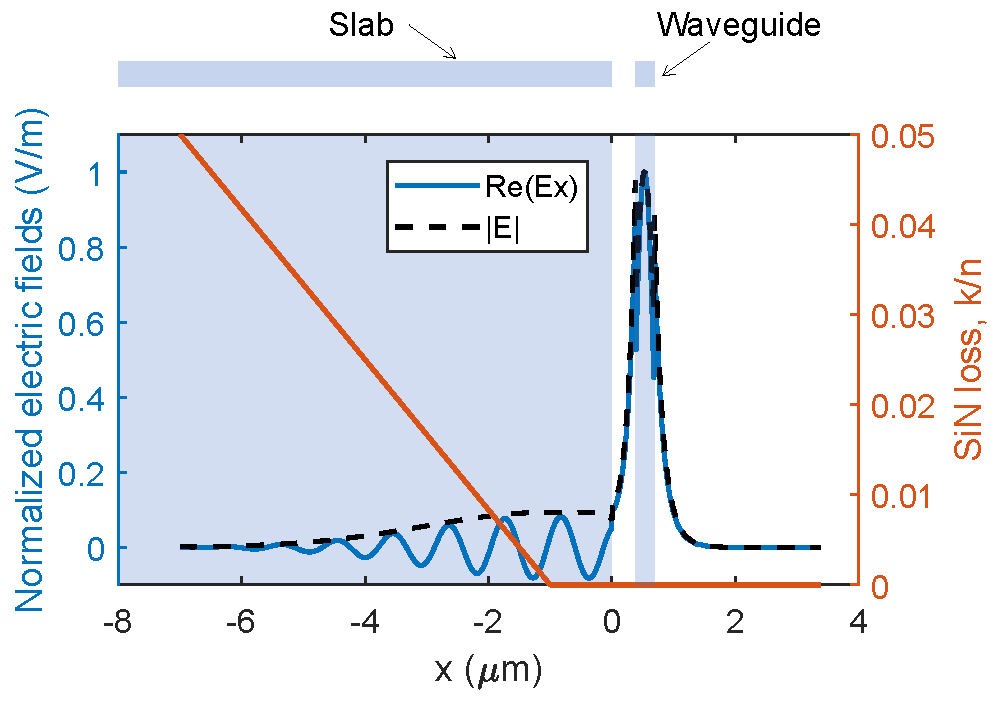


**FIG. S1.** Artificial loss profile (orange) and normalized electric fields through the center of the waveguide and slab: |E| (blue solid line) and Re(Ex) (black dashed line). The shaded regions represent SiN slab and waveguide. The loss is introduced far enough away from the gap (> 1 $\mu$m) to avoid affecting the result; the artificial loss (orange) linearly increases from zero loss at 1 $\mu$m away from the gap to the full value (loss tangent k/n=0.05) at the model domain wall, 7 $\mu$m away from the gap. The gradual increase ensures no reflection for the slab mode from the lossy region itself, and high enough final loss and large enough lossy region size to ensure no reflection from the model boundary can reach back to the coupling region. With this approach, results are independent of the specific choices of maximum loss tangent value, domain size or the location where loss is introduced (provided it is >1 $\mu$m from the gap). The decay in field profiles (blue line and black dashed line) due to the introduced artificial loss is evident. The fields are approaching zero on domain boundaries (at $x=-7 \mu m$). Note that the oscillation period in *x* (blue solid line) corresponds to the slab mode being launched at approximately 30 degrees to the waveguide, and therefore the period is about two times larger than the actual slab mode wavelength.
