# Supplementary figures and images for: Photonic waveguide to free-space Gaussian beam extreme mode converter
Source: Light Sci Appl. 2018 Oct 10;7:72. doi: 10.1038/s41377-018-0073-2 (PMC6177431; doi:10.1038/s41377-018-0073-2)

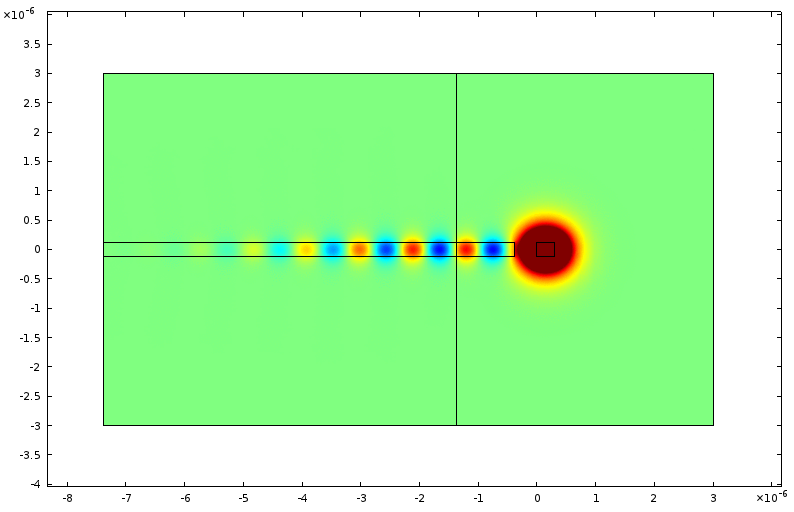

Supplement: Supplementary file 2 — Supplementary movie 1 [file 41377_2018_73_MOESM2_ESM.gif]
